# ICTV Virus Taxonomy Profile: *Redondoviridae*

**DOI:** 10.1099/jgv.0.001526

**Published:** 2020-12-01

**Authors:** Arwa Abbas, Louis J. Taylor, Ronald G. Collman, Frederic D. Bushman, ICTV Report Consortium

**Affiliations:** ^1^​ Department of Pathology and Laboratory Medicine, Children’s Hospital of Philadelphia, Philadelphia, PA, USA; ^2^​ Department of Microbiology, University of Pennsylvania Perelman School of Medicine, Philadelphia, PA, USA; ^3^​ Department of Medicine, University of Pennsylvania Perelman School of Medicine, Philadelphia, PA, USA

**Keywords:** *Redondoviridae*, ICTV Report, taxonomy, torbevirus, CRESS DNA virus

## Abstract

Viruses in the family *Redondoviridae* have a circular genome of 3.0 kb with three open reading frames. The packaged genome is inferred to be single-stranded DNA by analogy to related viruses. Redondoviruses were discovered through metagenomic sequencing methods in samples from human subjects and are inferred to replicate in humans. Evidence of redondovirus infection is associated with periodontitis and critical illness, but redondoviruses have not been shown to be the causative agent of any diseases. This is a summary of the International Committee on Taxonomy of Viruses (ICTV) Report on the family *Redondoviridae*, which is available at ictv.global/report/redondoviridae.

## Virion

The physical structure and properties of redondovirus particles are unknown. The capsid protein likely derives from the largest open reading frame (ORF). The number and orientation of capsomer units is unknown.

## Genome

**Fig. 1.**
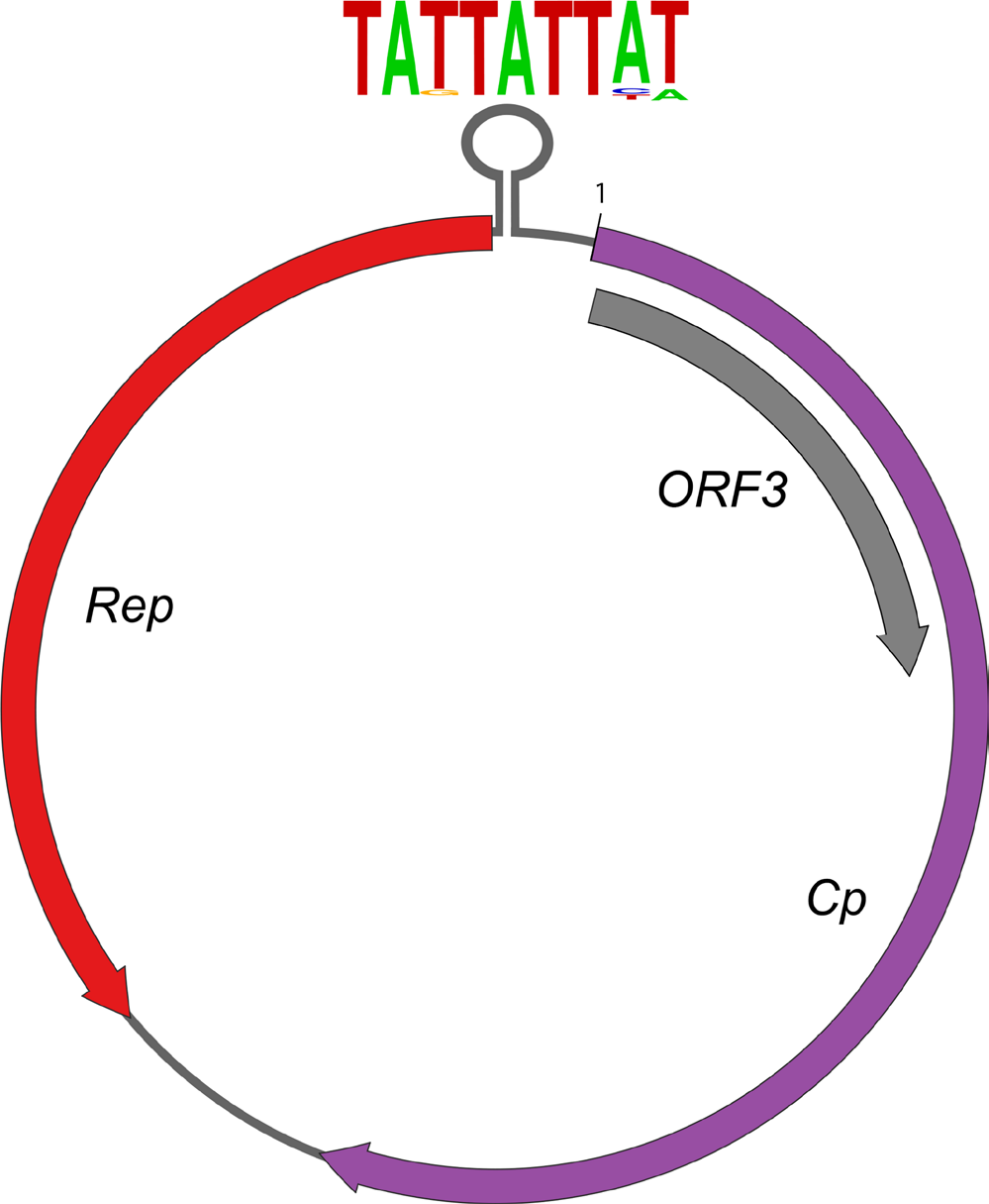
Redondovirus genome structure. Three ORFs (coloured arrows) flank a hairpin loop that contains the conserved nonanucleotide sequence shown.

The genome is circular and inferred to be ssDNA (Table 1) [1]. The genome has three ORFs, with the two largest ORFs in opposite orientations. One ORF (*Cp*) encodes the putative capsid and one (*Rep*) encodes the replication-associated protein (Rep). A third ORF, which overlaps *Cp*, encodes a protein with no homology to any known protein, but is conserved amongst all genome isolates. A stem–loop structure with a conserved nonanucleotide motif is found before the beginning of the *Rep* coding sequence (Fig. 1) [1] and is a likely candidate for the origin of replication, as in related circoviruses [2].

**Table 1.** Characteristics of members of the family *Redondoviridae*

Example:	human respiratory-associated brisavirus, isolate LC (KY052047), species *Brisavirus*, genus *Torbevirus*
Virion	Unknown
Genome	Circular 3.0 kb, inferred to be single-stranded DNA
Replication	Presumed to be by a rolling-circle mechanism
Translation	Unknown
Host range	Human
Taxonomy	Realm *Monodnaviria,* kingdom *Shotokuvirae,* phylum *Cressdnaviricota*, class *Arfiviricetes*, order *Recrevirales*; the genus *Torbevirus* includes two species

## Replication

Since redondoviruses do not encode a DNA polymerase, the replication cycle is presumed to be similar to the rolling-circle mechanism used by other circular DNA elements such as ssDNA viruses and bacterial plasmids. In this model, upon entry of a host cell and uncoating of the virion, the viral ssDNA genome is converted to dsDNA by host polymerases.The viral Rep protein binds in a sequence-specific manner to the stem–loop structure and nicks the dsDNA, creating a free 3′-hydroxyl end from which viral DNA synthesis can begin. The Rep protein, meanwhile, remains covalently bonded to the 5′-phosphate end. After one round of genome synthesis, the Rep protein releases one ssDNA genome, and the dsDNA template is regenerated for additional rounds of rolling-circle replication. Other details are unknown.

## Pathogenicity

Redondovirus nucleic acid sequences have been detected in both healthy humans and those with various diseases. The first reported genome was discovered in the respiratory tract of a febrile patient [3] who tested negative for a limited panel of other pathogens. Subsequently, full-length genomes were found in bronchoalveolar lavage from organ transplant donor lungs, lung transplant recipients and patients with sarcoidosis [4–6]. A large screen of metagenomic samples from humans, animals and the environment revealed that redondoviral genome sequences were only detected with reasonable certainty in human samples, primarily from the oro-respiratory tract, but also in the gut [1]. Detection by qPCR also revealed that redondovirus nucleic acid was present at relatively high levels in the upper and lower respiratory tract of patients in intensive care units, compared to healthy humans. Analysis of sequences [1] from two metagenomic studies of the oral cavity [7, 8] showed that the presence and abundance of redondoviral genome sequence was associated with periodontitis. Redondoviruses were also detected in sputum from a patient with respiratory symptoms in the absence of any identified respiratory pathogen [9].

## Taxonomy

Current taxonomy: ictv.global/taxonomy. The genus *Torbevirus* includes the species *Brisavirus* and *Vientovirus*.

## Resources

Current ICTV Report on the family *Redondoviridae*: ictv.global/report/redondoviridae
